# Athletes demonstrate an advantage in unconscious processing under working-memory load specifically within sport-related domains

**DOI:** 10.3389/fpsyg.2025.1697078

**Published:** 2025-11-12

**Authors:** Xuechen Mao, Qin Huang, Jilong Shi, Yanglan Yu, Anmin Li

**Affiliations:** 1 Department of Physical Education, Nanjing University of Chinese Medicine, Nanjing, China; 2 Department of Physical Education, Leshan Normal University, Leshan, China; 3 Department of Physical Education, Xiamen University, Xiamen, China; 4 School of Psychology, Shanghai University of Sport, Shanghai, China

**Keywords:** athletes, unconscious priming, working-memory load, executive attention, stimuli domain

## Abstract

A growing body of evidence indicates that unconscious priming requires attentional resources, including high-level cognitive resources such as executive attention. However, the generalizability of this finding remains unexplored. Specifically, it remains unclear whether unconscious priming in skilled athletes, who are widely considered to possess advantages in unconscious processing, is limited by executive attention, and whether such advantages transfer to non-experiential domains remains debated. This investigation seeks to address this research gap through two dual-task experiments utilizing a mixed experimental design, attempting to elucidate the characteristics of unconscious priming under working-memory load in experienced athletes. One hundred and twelve participants, half of whom were elite table tennis athletes, completed a dual-task paradigm combining an N-back task with a masked priming task. In Experiment 1, the stimuli in the priming task were unrelated to sports scenarios, whereas in Experiment 2, they were sport-related. Mixed-effects ANOVAs, incorporating within-subject, between-subject factors and between-experiment factor, were conducted to analyze behavioral data. The results showed that in Experiment 2, athletes displayed a significantly larger unconscious priming effect under working-memory load compared to non-athletes (η_p_^2^ = 0.08), whereas no such difference was observed in Experiment 1 (η_p_^2^ = 0.13). Moreover, across both experiments, the unconscious priming effect in athletes was impaired with increased working-memory load (η_p_^2^ = 0.17). Overall, these observations suggest that athletes excel in unconscious processing under working-memory load specifically in sports-related domains. Furthermore, athletes’ unconscious processing is limited by the available capacity of executive attention resources, which are occupied by increased working-memory load. Therefore, our study not only provides valuable insights into the boundary conditions of the athletes’ advantages in unconscious processing, but also extends extant frameworks of attention gating hypotheses of unconscious processing from non-athlete populations to elite athletes.

## 1 Introduction

Although we cannot perceive unconscious processing^1^, it exerts a crucial influence on our emotional (Gainotti, 2021; Tsikandilakis et al., 2021) and cognitive processes (Huang et al., 2023; Huang and Li, 2025). Consequently, substantial studies have been dedicated to exploring the characteristics and boundaries of unconscious information processing (Dehaene et al., 2006; Kouider and Dehaene, 2007; Mudrik and Deouell, 2022; Silva et al., 2018). The resource limitation systems of unconscious processing have long been a central topic in these discussions. Although early cognitive theories suggested that unconscious processing operates independently of cognitive resource limitations (Kouider and Dehaene, 2007; Moors and De Houwer, 2006), increasing evidence in recent years has claimed that unconscious processing requires attentional resources (Bahrami et al., 2007; Hung et al., 2020; Kiefer et al., 2019) and may even involve executive attention (Ansorge et al., 2014; Silverstein et al., 2015).

An increasing body of empirical evidence from behavioral and neural data strongly supports the notion that executive attention is not exclusive to the domain of consciousness but is also essential for unconscious processing. Earlier studies reported that unconscious priming depends on temporal attention (Fabre et al., 2007; Naccache et al., 2002), and found that temporal attention is modulated solely by the manipulation subsystem of working memory (Capizzi et al., 2012), rather than by its maintenance subsystem (Zanto et al., 2020). Several studies have also observed the involvement of the frontoparietal cortex in unconscious priming (Diao et al., 2021; Jiang et al., 2018; Shi et al., 2022; Ulrich et al., 2014). Our recent investigation, which directly assessed the effects of the two working memory subsystems on unconscious processing, indicated that unconscious priming decreased only with increased load on the manipulation subsystem, but not with increased load on the maintenance subsystem (Mao and Li, 2022; Mao et al., 2022).

Notably, the processing pathways for unconscious stimuli are flexible and context dependent, and can consequentially be enhanced through immediate (Kiefer and Martens, 2010; Kiefer, 2012; Ulrich and Kiefer, 2016) and long-tern experience (Geng et al., 2020; Güldenpenning et al., 2015; You et al., 2018). In the former case, processing pathways show transient sensitivity to subliminal stimuli due to previous conscious exposure, whereas in the latter, they demonstrate sustained sensitivity to subliminal stimuli resulting from professional training. To further investigate whether immediate experience also modulate the demand for executive attention in unconscious priming, that is, whether previously perceived stimuli modulate the effect of manipulation subsystem load on unconscious priming, we adopted a modified dual-task paradigm. We found that unconscious priming decreased with an increase in working-memory load when the dual-task paradigm shared a different stimuli domain, but not when the dual-task paradigm shared the similar stimuli domain (Mao et al., 2022). These observations suggest that neural pathways previously engaged in processing a specific type of supraliminal stimulus become more sensitive to the corresponding subliminal stimuli. Therefore, unconscious processing under working-memory load can be enhanced through immediate experience. However, it remains unclear whether unconscious processing under working-memory load can also be modulated by long-tern experience, such as specific training.

Athletes represent an excellent model for research in this domain, as their processing pathways have been proven to exhibit heightened sensitivity to subliminal stimuli due to their prolonged professional training (Geng et al., 2020; Güldenpenning et al., 2015; You et al., 2018). In sports scenarios, the rapid changes in the external environment often require responses that are too fast to rely on explicit discrimination of perceived motor information (Kibele, 2006). Indeed, the presentation duration of key motor information, such as the spin characteristics and trajectory of an approaching ball, is far below the visual threshold (Tamaki et al., 2024); thus, athletes, especially those from interactive sports (e.g., table tennis), are trained to respond rapidly and accurately based on their unconscious perception of the ball.

It should be pointed out that athletes’ sensitivity to subliminal stimuli is manifested not in their capacity to perceive the presence of such stimuli, but rather in their ability to process them in a resource-efficient manner. Adopting a combination of a masked priming paradigm with event-related potential (ERP) assessments, a recent study found that, compared to non-athletes, table tennis athletes showed decreased reaction times, enhanced unconscious priming effects, and remarkably reduced amplitude responses (Shi et al., 2024). This finding suggests that athletes process subliminal stimuli with relatively lower expenditure of attentional resources, aligning with numerous studies holding that specialized training in sports enables athletes to perform tasks with less neural activation, optimizing the utilization of attention resources (Li et al., 2023; Guo et al., 2017). Therefore, this raises the question of whether long-tern specialized training modulates the executive attention requirements of unconscious processing, specifically, whether athletes perform better unconscious processing abilities than non-athletes under manipulation subsystem load.

Research in this field typically employs the dual-task paradigm that combines a working-memory task with an unconscious processing task. We chose the N-back paradigm as the working-memory task because it occupies executive attention with increased load (Lilienthal et al., 2013; Meiron et al., 2013). The forward and backward masking priming paradigm was selected as the unconscious processing task due to its excellent suppression effects (Breitmeyer, 2015; Kouider and Dehaene, 2007). Furthermore, substantial studies have deployed this suppression method and identified experts’ advantage under unconscious conditions (Geng et al., 2020; Güldenpenning et al., 2015; Jiang et al., 2021). However, several findings indicated that athletes consumed more attention resources for processing unconscious stimuli although they manifested larger unconscious priming effects than non-athletes (Jiang et al., 2024; Meng et al., 2022; You et al., 2018). It is worth noting that the unconscious stimuli utilized in these studies varied significantly, ranging from arrows or simple geometric shapes unrelated to sport-specific scenarios, to movements patterns of bodies or balls related to sporting contexts. A recent review of multilevel meta-analysis evaluated 11 prospective-design studies and concluded that stimuli domain modulates athletes’ performance in cognitive function tests, with athletes performing better under sport-specific stimuli compared to general ones (Kalén et al., 2021). Considering that the stimuli domain may affect the attentional resource requirements during unconscious processing in athletes, we conducted two experiments to investigate this issue. In addition, to preclude any moderating effect of immediate experience on unconscious priming under working-memory load (Mao and Li, 2022; Mao et al., 2022), the N-back task did not share a similar stimuli domain with the masked priming task. Thus, the present work followed our previously designed dual-task paradigm, which combines an N-back task with a sandwich masking task to test whether athletes exhibit a stronger unconscious priming effect relative to non-athletes under working-memory load. We selected table tennis players as participants because a growing body of evidence claims that skilled table tennis athletes demonstrate enhanced unconscious priming effects than non-athletes (Guo et al., 2017; Shi et al., 2024; Wei and Li, 2017).

In general, what previous studies have not addressed is whether athletes demonstrate an advantage in unconscious processing under working-memory load and whether stimuli domain affects this advantage. To solve these issues, the current study was a 2 (training experience of sports) × 2 (level of working-memory load) × 2 (sports relevance of unconscious stimulus) × 2 (congruency condition of unconscious stimulus) design (see section “2 Materials and methods”). The investigation was divided into two experiments in order to reduce the overall experimental time that participants would experience, which in turn would decrease a serious risk of mental fatigue, reduced motivation, and attentional decline over time. A total of 200 volunteers signed up for the mixed-design experiment, and 112 students finally participated in the experiment according to the inclusion criteria (half of whom were professional table tennis players). The 56 athletes and 56 non-athletes were randomly divided into Groups A and B. Group A (28 athletes and 28 non-athletes) participated in Experiment 1, and Group B (the other 28 athletes and 28 non-athletes) participated in Experiment 2. Experiment 1 employed geometric shapes as prime and target stimuli in the masked priming task to exam whether athletes would display an advantage in unconscious priming under working-memory load when unconscious processing was domain-general. Experiment 2 introduced balls with hitting spot as prime and target stimuli in the suppressed priming task to explore whether athletes such an advantage would emerge when unconscious processing was domain-specific. Based on existing literature, we hypothesized that athletes could exhibit better unconscious priming effects under working-memory load compared to non-athletes when unconscious processing occurred in a sport-specific context as opposed to a general one. We also anticipated that unconscious priming effects would decrease as increased working-memory load in both athletes and non-athletes.

## 2 Materials and methods

This was a mixed experimental design. The independent variables include between-subjects variables (athletes vs. non-athletes), within-subjects variables, including memory load (high vs. low) and consistency (consistent vs. inconsistent), and between-experiment variable (sports unrelated vs. sports related). The main dependent variables are response time (RT) and response accuracy (RA).

### 2.1 Experiment 1

We first tested the unconscious priming performance of athletes under working-memory load when the unconscious stimuli were unrelated to their expertise domain. Based on prior literature (Kalén et al., 2021; Mao and Li, 2022), we hypothesized that there would be no significant difference in unconscious priming effects with increased working-memory load between athletes and non-athletes.

#### 2.1.1 Participants

Participants were recruited through online advertisements at Shanghai University of Sport and were anonymous and volunteered on to be included in this study. The inclusion criteria required the participants to have normal or corrected-to-normal vision and no psychological or neurological disorders. For the table tennis athletes, eligibility required intensive training in table tennis with more than 8 years and achievement of at least the second level as per the national standard in China. The non-athlete group consisted of participants without any formal practical experience in the sport domain or any competitive level certificate.

Based on our prior empirical studies (Mao and Li, 2022; Mao et al., 2022), power analysis (G*Power3.1, α = 0.05, power = 0.95, effect size = 0.25) showed that a minimum of 36 participants are needed. To further enhance the statistical robustness, we ultimately recruited 56 participants. The experimental group consisted of 28 elite table tennis athletes (14 men; 3 left-handed; mean age, 20.46 ± 0.34), who were selected due to their extensive table tennis practice. The comparison group was composed of 28 non-athletes (14 men, 1 left-handed; mean age, 19.89 ± 0.46), who were selected for their lack of experience in table tennis or any other sports experience. There was no significant difference in age between the two groups of participants [t_(54)_ = 1.01, *p* = 0.32]. The athletes experienced table tennis training with an average of 9.42 ± 1.18 years. The majority of nonathletes were first and second-year students majoring in Sports English, Sports News, and related fields. All participants provided written informed consent, and all participation was approved by the Ethical Committee of Shanghai University of Sport (No. 102772021RT020). Additionally, the participants confirmed that they had not participated in similar experiments before this study.

#### 2.1.2 Materials

Regarding the unconscious priming tasks, an ellipse (vertical × horizontal: 1.5° × 4.0°) and a diamond (4.0° × 1.5°) were employed as targets, and four ellipses (varying from 1.1° × 4.0° to 1.9° × 4.0°) and four diamonds (varying from 4.0° × 1.1° to 4.0° × 1.9°) that were proportionally enlarged or narrowed compared with the target size were utilized as primes. The forward and backward masks (4.0° × 4.0°) were drawn with many randomly oriented characters. For the N-back tasks, each memory item was an Arabic number from zero to nine (1.0° × 0.7°) randomly presented in the center of the screen. Altogether, 8 primes, 2 targets, 2 masks, and 10 memory items were depicted in white on a dark gray screen with an approximate 70 cm viewing distance. The experiment was conducted deploying a computer (resolution = 1,400 × 900 pixels, frequency = 60 Hz, and frame duration = 16.67 ms) with an E-prime 2.0 software package (Schneider et al., 2002). The luminance output of the monitor was linearized and calibrated using a Minolta LS-110 photometer. The background luminance was set to 10 cd/m^2^ and the luminance of the stimulus was 60 cd/m^2^. The Michelson contrast of the stimulus, calculated as (Lmax−Lmin)/(Lmax+Lmin), was 71.42%.

#### 2.1.3 Procedure

##### 2.1.3.1 Main task

**FIGURE 1:**
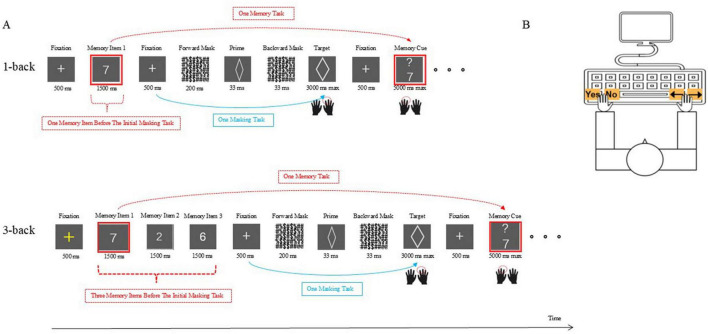
Schematic illustration of the dual-task paradigm in Experiment 1. **(A)** An example of the first N-back task combined with the initial unconscious priming task under low load (top) or under high load (bottom). **(B)** A schematic illustration of the key press during the dual-task paradigm. The only variation was that in Experiment 2, the stimuli in the unconscious priming task were table tennis balls with hitting points.

Our main task was developed by combining an N-back task with an unconscious priming task. That is, each forward-backward masking priming task was inserted into each N-back task (see Figure 1). Participants were required to remember the memory items and update them as instructed (an N-back task using numbers as stimuli) while making judgments on geometric figures (a masked priming task using geometric figures as stimuli).

Each memory trial began with a yellow central fixation dot (500 ms) followed by a memory item for 1,500 ms under the low-load condition (three memory items for 4,500 ms with each displaying sequentially for 1,500 ms under the high-load condition). During the item showing, participants had to maintain it because they were to decide whether a newly presented item was identical to the previously memorized item (whether a new item was identical to the first memory item under the high-load condition) after completing one masked priming task. Thereafter, a white fixation cross was displayed for 500 ms to indicate that one masked perceptual priming task was going to come.

In the unconscious priming task, after the white fixation, participants first viewed a forward mask with a 200 ms duration, which was replaced by a prime shape that appeared for 33 ms, after which a backward mask was shown for 33 ms. Then, the target shape appeared for 3,000 ms until a response was made. Participants decided whether or not the target shape was a diamond or ellipse and reported their decision by pressing one of two response buttons with their right hands (left arrow for diamond and right arrow for ellipse).

After the priming task was completed, a white fixation cross (500 ms) was shown, followed by a memory cue. The participants had to indicate whether the new item matched the one (the first one at high load condition) that came one masking task before it. Participants were instructed to not only respond to the new memory cue with their left hand (“z” for “yes” and “c” for “no”) but also maintain the new memory cue. This was because the new memory cue would be compared with the next memory cue after the completion of the subsequent next masking task (or after the completion of three masking tasks under the high-load condition). When a response was collected or after 5,000 ms, the screen went blank, and the next masking task was to start.

Altogether, the main task consisted of 400 masked priming tasks and 400 working memory tasks presented in 40 dual-task trials (20 trials for each load condition). At each load condition, ten memory tasks were intermixed with ten priming tasks in one trial, and participants were asked to give responses as correctly and as fast as possible. The order of the memory task and the priming task was fixed and the inter-trial intervals was fixed. For mitigating sequence effects, the order of the load conditions was counterbalanced across the participants. Within each load condition, in half the trials, the prime was congruent with the target while the prime was incongruent for the other half. Additionally, the two shapes appeared equally often as primes and as targets. Additionally, each memory item appeared equally often. After completing each trial, the participants would be asked whether they need a break. Once the participant had rested sufficiently, they pressed the spacebar on the computer to proceed to the next trial.

Prior to the main task, a training phase of 60 single priming tasks, 60 single memory tasks and 60 dual-task trials (30 for each load condition) needed to be done. It should be pointed out that training-phase data were used solely for familiarization and excluded from all inferential analyses.

##### 2.1.3.2 Identification task

After the main task, participants were informed about the presence of the masked primes, and then they had to complete an identification task consisting of objective and subjective measures. The structure of the identification task was similar to that of the main task to keep the stimulation comparable. In each trial, the participants were asked to identify the prime between the two masks and to perform the prime decision with the same response categories as in the main task. Following the button press, a short version of the 4-point perceptual awareness scale (PAS) was shown. The observers had to choose one of the following options to rate the subjective visible levels of the masked prime: 1 = No experience, 2 = Weak glimpse, 3 = Almost clear, 4 = Absolutely clear. Regarding the memory part, we selected the low-load condition only because the suppression effect under low load was reduced compared with that under high load (Todd et al., 2005). Participants had to undergo 20 practice trials before initiating the identification task, which consisted of 100 identification trials, which contained 100 memory tasks and 100 subjective and 100 objective tasks. The participants completed the task carefully and thoroughly with unlimited time, ensuring accuracy as the priority.

#### 2.1.4 Analysis

##### 2.1.4.1 Identification task

For objective measures, RA was assessed for significant deviation from 50% with a one-sample *t*-test. As a measure for prime identification, the signal detection measure d’ was calculated by transforming the respective false alarm rate (erroneous responses to incongruent trials) into a z score, which was subtracted from the z score pertaining to the hit rate (correct responses to congruent trials). The subject-wise d’ values were assessed for significant deviation from zero using one-sample t tests. Data from participants who reported having seen the prime or RA from objective measures significantly exceeding chance performance were discarded. Furthermore, a Pearson’s correlation between the magnitude of the masked priming effect under low load and d’ was performed to rule out the possibility that masked priming correlated with prime visibility.

##### 2.1.4.2 Main task

The load condition (low vs. high) of the working memory task, congruency condition (congruent vs. incongruent) of the masked priming task, and group type were the independent variables (the former two were within-subjects factors, and the latter was a between-subjects factor), whereas RT, RA and subliminal priming (the RT on incongruent trials minus the RT on congruent trials) of the priming task as well as RT and RA of the memory task were dependent variables. Individual means and standard deviations of the correct responses were first computed for each of the dependent variables. Data from participants were excluded when their RA was more than 2 standard deviations below the group average. Furthermore, trials whose RTs were faster or slower than two standard deviations of the individual mean were rejected as outliers. The statistical analyses were performed using SPSS 25.0. Mixed-effects ANOVAs were conducted, incorporating both within-subject and between-subject factors, to analyze behavioral data from memory and priming tasks. To ensure the statistical accuracy and validity of the interpretation of results, the fundamental statistical assumptions prior to applying the ANOVA were tested, such as homogeneity of variance. The degrees of freedom were adjusted using the Greenhouse–Geisser correction for non-sphericity, and the *post-hoc* tests underwent Bonferroni correction as needed.

#### 2.1.5 Results

Four athletes and three non-athletes were excluded from the analysis due to significantly higher RA in the objective visibility test relative to the chance level and/or significantly lower RA in the N-back task compared with the average level of their respective group. The remaining 24 athletes and 25 non-athletes were included in further analysis. The *p*-values for the homogeneity of variance of all dependent variables were greater than 0.05.

##### 2.1.5.1 Identification task

Our sandwich masking was effectual. No one reported having seen the prime (mean ± standard error of the subjective measure for athletes, 1.39 ± 0.11; for non-athletes, 1.42 ± 0.08, Supplementary Figure 1). No significance was found between the RA of the objective measure for the two groups and the chance level for athletes (47.80 ± 1.85%), *t*_(23)_ = −1.19, *p* = 0.25, or for non-athletes (48.93 ± 1.97%), *t*_(24)_ = −0.54, *p* = 0.59. Moreover, *d*’ of athletes (−0.04 ± 0.07) and non-athletes (0.00 ± 0.08) did not differ statistically from zero, *t*_(23)_ = −0.61, *p* = 0.55, *t*_(24)_ = 0.03, *p* = 0.98, respectively. Additionally, there was no significant difference between athletes and non-athletes in subjective measure, *t*_(47)_ = −0.18, *p* = 0.86, or objective measure, *t*_(47)_ = −0.42, *p* = 0.68, and *d*’, *t*_(47)_ = −0.41, *p* = 0.68.

Furthermore, the distribution of d’ from the two groups (athletes: Kolmogorov–Smirnov = 0.10, *p* = 0.20; non-athletes: Kolmogorov–Smirnov = 0.10, *p* = 0.20) and the distribution of unconscious priming from the athletes (Kolmogorov–Smirnov = 0.13, *p* = 0.20) were normal. The distribution of unconscious priming from the non-athletes (Kolmogorov–Smirnov = 0.20, *p* = 0.01) was not normal and was converted logarithmically before the correlation analysis. No correlation between d’ and unconscious priming was found, r_(24)_ = −0.07, *p* = 0.79, r_(25)_ = 0.08, *p* = 0.74, for athletes and non-athletes, respectively. These results supported that unconscious priming of the two groups was not the result of their awareness of the masked primes.

##### 2.1.5.2 Main task

###### 2.1.5.2.1 Memery task

The analysis of RT and RA of the memory task suggested that load manipulation was efficacious. For RT (athlete outliers for low and high load: 2.50%, 6.01%; non-athletes: 1.60%, 7.08%), there was a main effect of load, *F*_(1,47)_ = 39.97, *p* < 0.001, η_p_^2^ = 0.46, indicating that RT at low load was significantly shorter than that at high load. Furthermore, a significant main effect of group was found, *F*_(1,47)_ = 8.89, *p* < 0.01, η_p_^2^ = 0.16, suggesting that athletes reacted faster than non-athletes.

**FIGURE 2:**
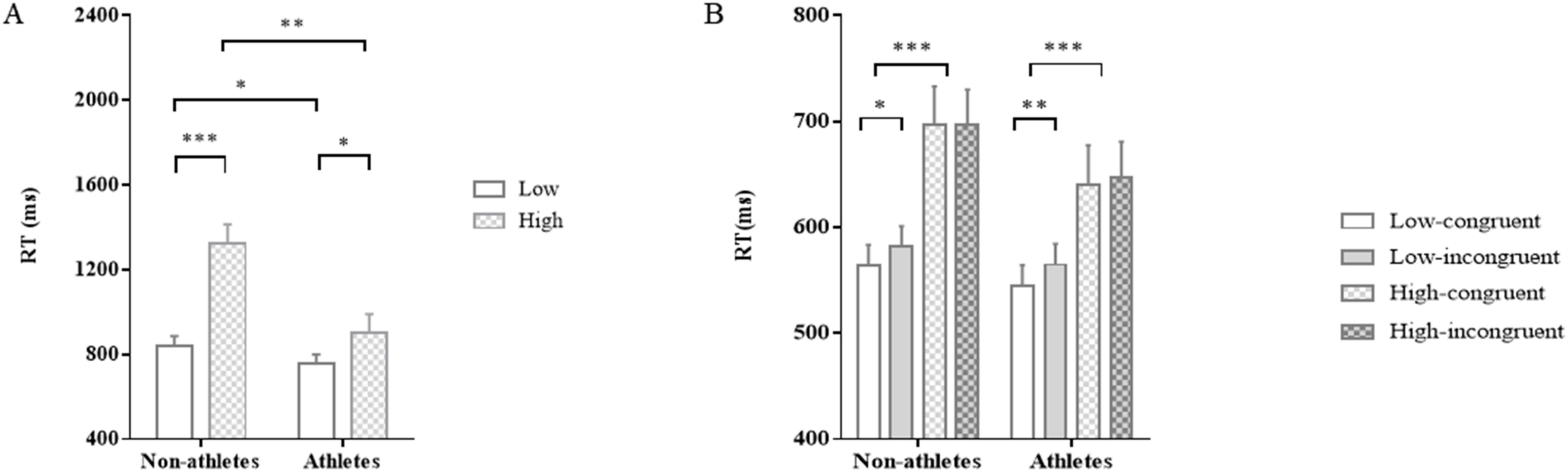
The results of Experiment 1. Response times in the two groups for the N-back task **(A)** and the unconscious priming task **(B)**. The error bars denote standard error of the mean (SEM). *p < 0.05, **p < 0.01, ***p < 0.001.

Importantly, we observed an interaction between group and load conditions, *F*_(1,47)_ = 11.54, *p* = 0.001, η_p_^2^ = 0.20 (Figure 2A). *Post-hoc* comparisons showed that athletes did not perform faster relative to non-athletes at low load, *p* = 0.17, whereas athletes performed significantly faster than non-athletes at high load, *p* = 0.001.

Regarding RA, a main effect of load was found, *F*_(1,47)_ = 15.92, *p* < 0.001, η_p_^2^ = 0.25, suggesting that RA at low load was significantly higher than that at high load. Additionally, neither a main effect of group, *F*_(1,47)_ = 0.24, *p* = 0.63, η_p_^2^ < 0.001, nor an interaction between load and group conditions, *F*_(1,47)_ = 0.84, *p* = 0.37, η_p_^2^ = 0.02, was found. These data indicated that regardless of athlete or non-athlete status, RA at low load was always greater than that at high load in the memory task.

###### 2.1.5.2.2 Masked priming task

For RT (athlete outliers for low and high load: 1.96%, 5.71%; non-athletes: 1.94%, 6.99%), there was a main effect of load, *F*_(1,47)_ = 38.91, *p* < 0.001, η_p_^2^ = 0.45 (see Table 1). This indicated that load manipulation was efficacious because RT at low load was significantly shorter than that at high load. Furthermore, we observed a strong unconscious priming effect, that is, a main effect of congruency, *F*_(1,47)_ = 9.78, *p* < 0.01, η_p_^2^ = 0.17, suggesting that RT on congruent trials was significantly shorter than that on incongruent trials. However, there was no main effect of group, *F*_(1,47)_ = 0.97, *p* = 0.33, η_p_^2^ = 0.02. It should be pointed out that athletes seemed to react faster than non-athletes in the unconscious priming task, although this did not reach a significant level.

**TABLE 1:** Mean reaction times (RTs in milliseconds), and error rates (RAs in percentage) as a function of working-memory load (1-back versus 3-back), and congruency conditions (congruent versus incongruent) in Experiment 1.

			Experiment 1			
			1-back		3-back	
			A	N	A	N
WMT	RT		755.07 ± 44.64	841.72 ± 43.74	900.92 ± 89.85	1,326.33 ± 88.03
	RA		998.41 ± 0.30	98.26 ± 0.30	90.08 ± 3.00	91.21 ± 3.10
UPT	RT	C	544.47 ± 19.81	564.04 ± 19.40	640.53 ± 37.08	697.03 ± 36.33
		I	564.64 ± 19.81	581.65 ± 19.41	646.90 ± 34.08	696.83 ± 33.39
	RA	C	98.80 ± 0.30	98.60 ± 0.30	99.40 ± 0.20	99.20 ± 0.20
		I	98.50 ± 0.30	98.60 ± 0.30	99.30 ± 0.20	98.60 ± 0.40

WMT, working memory task; UPT, unconscious priming task; RT, response time; RA, response accuracy; C, congruent condition; I, incongruent condition; A, athletes; N, non-athletes. Mean ± standard error.

Most importantly, there was a significant interaction between load and congruency, *F*_(1,47)_ = 6.72, *p* = 0.01, η_p_^2^ = 0.13 (Figure 2B). Simple effects analysis reported that at low load, RT in congruent condition was significantly shorter than that in incongruent condition (*p* < 0.001), whereas at high load, there was no significant difference in RT between congruent and incongruent conditions (*p* = 0.47). In addition, neither an interaction between load and group conditions, *F*_(1,47)_ = 1.05, *p* = 0.31, η_p_^2^ = 0.02, nor an interaction between congruency and group conditions, *F*_(1,47)_ = 0.35, *p* = 0.56, η_p_^2^ < 0.01, was statistically significant. Moreover, no three-way interaction of group factor, load factor, and congruency factor was found, *F*_(1,47)_ = 1.10, *p* = 0.30, η_p_^2^ = 0.02, indicating that all participants experienced impaired unconscious performance when working memory was loaded.

Regarding RA, neither the main effect of group, *F*_(1,47)_ = 0.50, *p* = 0.48, η_p_^2^ = 0.01, or congruency condition, *F*_(1,47)_ = 0.50, *p* = 0.48, η_p_^2^ = 0.01, nor load condition, *F*_(1,47)_ = 3.10, *p* = 0.09, η_p_^2^ = 0.06, was observed. Similarly, no significant interaction was identified.

#### 2.1.6 Discussion

The results of Experiment 1 showed that when unconscious stimuli were general and sport-unrelated, there was no significant difference in the unconscious priming effect between athletes and non-athletes under working memory load. Moreover, the unconscious priming effect decreased significantly with an increase in working-memory load. These observations are inline with our prior findings, suggesting that unconscious priming shares a common resource pool with the manipulation subsystem (Mao and Li, 2022; Mao et al., 2022). In those studies, participants performed a modified dual-task paradigm intermixing an N-back task with a masked shape discrimination task. The results exhibited an increase in manipulation load decreased the magnitude of unconscious priming effects, whereas an increase in maintenance load did not affect unconscious priming. Given that increased manipulation load occupies executive attention (Baddeley, 2012; Chow and Conway, 2015; Engle, 2018; Lenartowicz et al., 2021), these observations from Experiment 1, together with those earlier findings, provide strong evidence to support that unconscious processing requires executive attention and is impaired by enhanced working-memory load.

It is important to stress that, different from our earlier studies, the current work is the first to explore the relationship between unconscious priming and attentional load through incorporating skilled tablet tennis athletes as participants. These athletes are widely identified to be sensitive to subliminal stimuli (Geng et al., 2020; Meng et al., 2019, 2022; You et al., 2018) and capable of performing cognitive processing with minimal resource expenditure (Guo et al., 2017; Shi et al., 2024; Wei and Li, 2017, 2018). Therefore, the present article extends the attention limitation hypothesis of unconscious processing to a broader population.

Given that the stimuli adopted in the masked priming task in Experiment 1 were domain-general, which might affect athletes’ unconscious processing under working-memory load (Kalén et al., 2021; Kiesel et al., 2009), Experiment 2 utilized domain-specific stimuli as subliminal primes and targets to further test whether motor expertise would modulate unconscious priming under working-memory load.

### 2.2 Experiment 2

Experiment 2 assessed whether athletes would exhibit superior unconscious performance with increased manipulation load when the unconscious stimuli were related to their expertise domain. Based on prior literature (Kalén et al., 2021; Shi et al., 2024), we hypothesized a significant effect of group, suggesting that athletes would demonstrate larger unconscious priming effects than non-athletes under increased working-memory load.

#### 2.2.1 Participants

The participant allocation method used in Experiment 2 were identical to those used in Experiment 1. After selection, twenty-eight athletes (14 men; 2 left-handed; mean age, 20.14 ± 0.44) and non-athletes (14 men; 1 left-handed; mean age, 20.43 ± 0.42) participated in Experiment 2. Comparably, there was no significant difference in age between the two groups of participants [t_(54)_ = −0.47, *p* = 0.64]. The recruited athletes experienced table tennis training with an average of 9.28 ± 1.28 years. The majority of nonathletes were first and second-year students majoring in Sports English, Sports News, and related fields. Additionally, the participants confirmed that they had not participated in similar experiments before this study. Additionally, the *p*-values for the homogeneity of variance of all dependent variables were greater than 0.05.

#### 2.2.2 Materials, procedure

**FIGURE 3:**
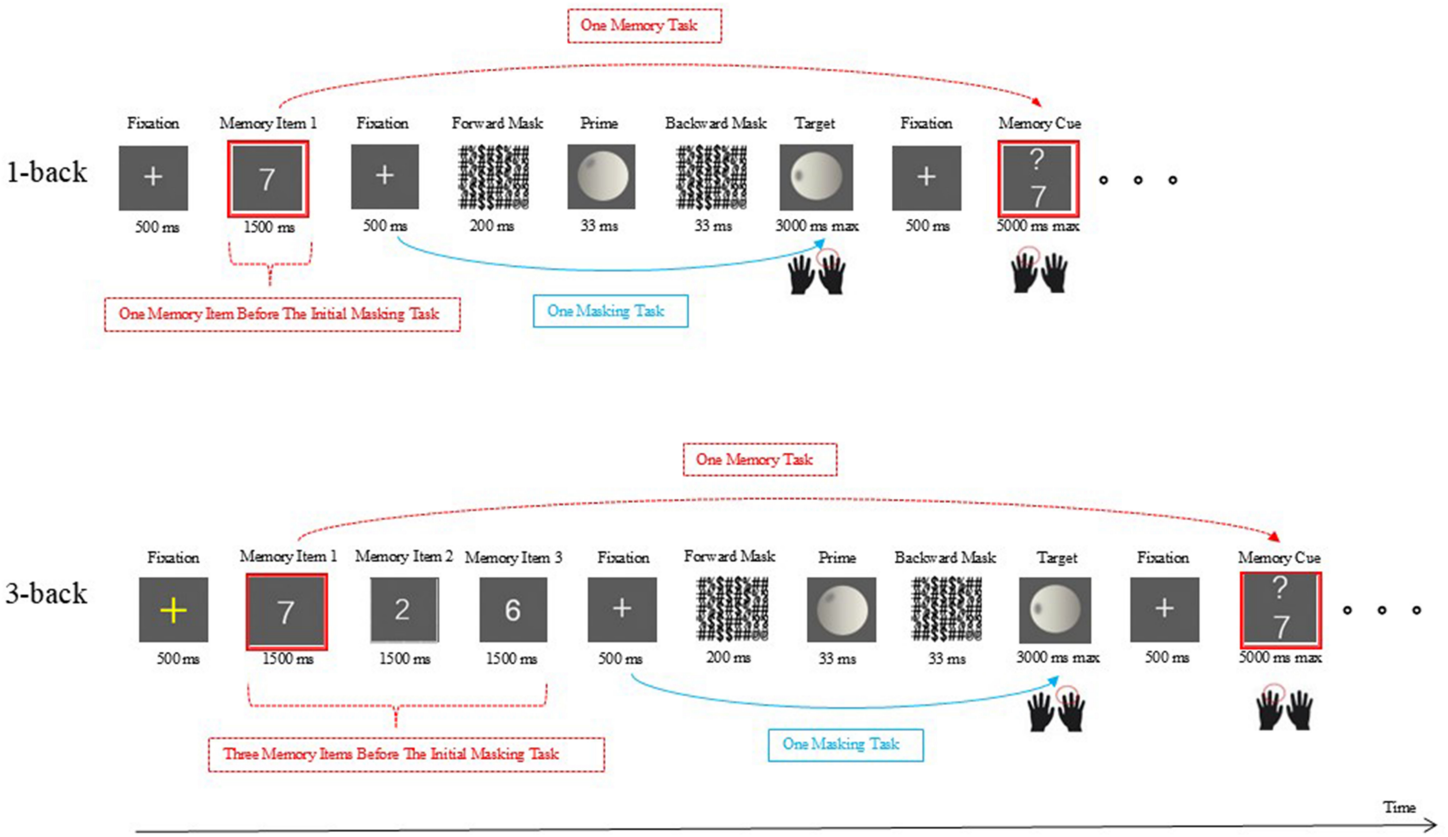
Schematic illustration of the dual-task paradigm in Experiment 2.

The materials and procedure used in Experiment 2 were identical to those used in Experiment 1, except that the prime and target stimuli employed in the unconscious priming task switched from general geometric shapes to table tennis balls with hitting points, which were related to sport-specific scenarios (Figure 3). Based on the principles of kinematics and sports biomechanics, key elements such as hitting point were selected, and simulated table tennis images with information structures of the hitting area were created using 3ds Max 2012 software. The ball surface was marked with dark gray shadows at angles of 15°, 30°, 45°, 60°, 75°, 105°, 120°, 135°, 150°, and 165° as ball’s hitting points, resulting in a total of 5 rendered 3D images of the balls with hitting points on the left side and 5 images of the balls with hitting points on the right side. These 10 balls (2.0° × 2.0°) with hitting points thus served as the stimulus materials for the masked priming task. Among them, balls with hitting points at angles of 45° and 135° used as target stimuli, while the remaining ball images functioned as masked primes in the unconscious priming task.

In the unconscious priming tasks, participants were instructed to judge the orientation of the hitting point and reported their judgment by pressing one of two response buttons with their right hands (left arrow for the hitting points on the left side and right arrow for the hitting points on the right side). Therefore, the congruency condition was determined by whether the prime and target stimuli required the same response.

#### 2.2.3 Analysis

The analysis for Experiment 2 follows that of Experiment 1. We also compared the differences in the performance of the identification and the main task between Experiments 1 and 2.

#### 2.2.4 Results

Three athletes were excluded from the analysis due to their significantly lower RA in the N-back task compared with the average level of the athletes group. The remaining 25 athletes and 28 non-athletes were included in further analysis. Additionally, there was no significant difference in age among participants between the two experiments [t_(100)_ = 0.16, *p* = 0.87].

##### 2.2.4.1 Identification task

Masking method was equally useful. No participant reported having seen the prime (athletes, 1.43 ± 0.10; non-athletes, 1.51 ± 0.09, Supplementary Figure 2). No significance was found between the RA of the objective measure for the two groups and the chance level for athletes (49.83 ± 1.13%), *t*_(24)_ = −0.15, *p* = 0.88, or for non-athletes (51.54 ± 0.97%), *t*_(27)_ = 1.59, *p* = 0.12. Moreover, *d*’ of athletes (−0.02 ± 0.05) and non-athletes (−0.06 ± 0.06) did not differ statistically from zero, *t*_(24)_ = −0.34, *p* = 0.74, *t*_(27)_ = −1.05, *p* = 0.30, respectively. Additionally, there was no significant difference between athletes and non-athletes in subjective measure, *t*_(51)_ = 0.05, *p* = 0.95, or objective measure, *t*_(51)_ = −1.15, *p* = 0.25, and *d*’, *t*_(51)_ = 0.59, *p* = 0.56.

Furthermore, the distribution of *d*’ from the two groups (athletes: Kolmogorov–Smirnov = 0.11, *p* = 0.20; non-athletes: Kolmogorov–Smirnov = 0.08, *p* = 0.20) and the distribution of unconscious priming from the two groups (athletes: Kolmogorov–Smirnov = 0.17, *p* = 0.06; non-athletes: Kolmogorov–Smirnov = 0.15, *p* = 0.12) were normal. No correlation between *d*’ and unconscious priming was found, *r*_(25)_ = −0.01, *p* = 0.97, *r*_(28)_ = −0.29, *p* = 0.13, for athletes and non-athletes, respectively. These results supported that unconscious priming of the two groups was not the result of their awareness of the masked primes.

Moreover, there was no significant difference between the two experiments in subjective measure, t_(100)_ = 0.77, *p* = 0.45, or objective measure, t_(100)_ = 1.57, *p* = 0.12, and d’, t_(100)_ = −0.34, *p* = 0.73.

##### 2.2.4.2 Main task

###### 2.2.4.2.1 Memery task

**FIGURE 4:**
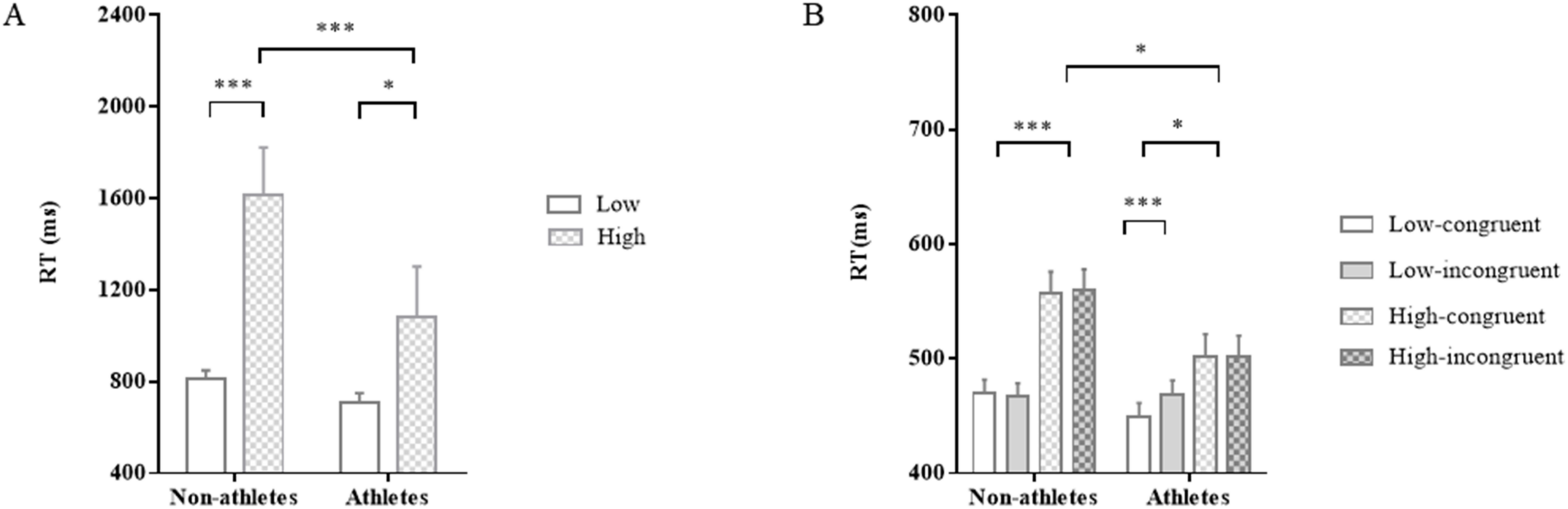
The results of Experiment 2. Response times in the two groups for the N-back task **(A)** and the unconscious priming task **(B)**. *p < 0.05, ***p < 0.001.

The analysis of RT and RA of the memory task suggested that load manipulation was effectual. For RT (athlete outliers for low and high load: 2.98%, 6.42%; non-athletes: 1.16%, 7.11%), there was a main effect of load, *F*_(1,51)_ = 19.11, *p* < 0.001, η_p_^2^ = 0.27, indicating that RT at low load was significantly shorter than that at high load (Figure 4A) and (Table 2). No main effect of group was found, *F*_(1,51)_ = 3.50, *p* = 0.07, η_p_^2^ = 0.06, although athletes seemed to react faster than non-athletes. No interaction between load and group conditions was observed, *F*_(1,51)_ = 2.54, *p* = 0.12, η_p_^2^ = 0.05.

**TABLE 2:** Mean reaction times (RTs in milliseconds), and error rates (RAs in percentage) as a function of working-memory load (1-back versus 3-back), and congruency conditions (congruent versus incongruent) in Experiment 2.

			Experiment 2			
			1-back		3-back	
			A	N	A	N
WMT	RT		708.11 ± 41.61	809.87 ± 39.32	1,083.43 ± 218.77	1,615.54 ± 206.72
	RA		97.60 ± 0.70	95.80 ± 0.70	93.60 ± 1.30	95.40 ± 1.20
UPT	RT	C	448.89 ± 12.31	470.02 ± 11.63	501.69 ± 19.66	557.4 ± 18.58
		I	468.48 ± 12.53	466.93 ± 11.84	501.47 ± 18.79	560.19 ± 17.76
	RA	C	99.70 ± 0.30	98.80 ± 0.30	99.40 ± 0.20	99.20 ± 0.20
		I	99.00 ± 0.30	98.40 ± 0.30	99.30 ± 0.20	98.60 ± 0.20

WMT, working memory task; UPT, unconscious priming task; RT, response time; RA, response accuracy; C, congruent condition; I, incongruent condition; A, athletes; N, non-athletes. Mean ± standard error.

Regarding RA, a main effect of load was found, *F*_(1,51)_ = 6.38, *p* = 0.01, η_p_^2^ = 0.11, suggesting that RA at low load was significantly higher than that at high load. Furthermore, we observed an interaction between group and load conditions, *F*_(1,51)_ = 4.15, *p* = 0.02, η_p_^2^ = 0.08. *Post-hoc* comparisons showed that there was no significant difference in RA between two load conditions for non-athletes, *p* = 0.72, whereas athletes’ RA at low load was significantly greater than that at high load, *p* < 0.01. Additionally, we did not observe a main effect of group, *F*_(1,51)_ < 0.001, *p* = 1.00, η_p_^2^ < 0.001.

Moreover, neither a main effect of experiment, *F*_(1,98)_ = 1.02, *p* = 0.32, η_p_^2^ = 0.01, *F*_(1,98)_ = 0.03, *p* = 0.86, η_p_^2^ < 0.001, nor an interaction between load and experiment conditions, *F*_(1,98)_ = 3.44, *p* = 0.07, η_p_^2^ = 0.03, *F*_(1,98)_ = 0.03, *p* = 0.87, η_p_^2^ < 0.001, nor a three-way interaction among load, group, and experiment conditions, *F*_(1,98)_ = 0.10, *p* = 0.80, η_p_^2^ = 0.001, *F*_(1,98)_ = 2.57, *p* = 0.11, η_p_^2^ = 0.03, was found in RT and RA.

###### 2.2.4.2.2 Masked priming task

For RT (athlete outliers for low and high load: 2.19%, 5.93%; non-athletes: 1.86%, 7.01%), there was a main effect of load, *F*_(1,51)_ = 52.58, *p* < 0.001, η_p_^2^ = 0.51. This indicated that load manipulation was useful because RT at low load was significantly shorter than that at high load. Importantly, there was a significant interaction between load and group, *F*_(1,51)_ = 6.66, *p* = 0.01, η_p_^2^ = 0.12 (see Table 1). *Post-hoc* comparisons held that at low load, there was no significant difference in RT between the two groups (*p* = 0.57), whereas at high load, RT of athletes was significantly shorter than that in of non-athletes (*p* = 0.03).

It should be pointed out that a significant interaction between group and congruency was observed, *F*_(1,51)_ = 4.63, *p* = 0.04, η_p_^2^ = 0.08. *Post-hoc* comparisons revealed that athletes’ RT in congruent trials was significantly shorter than that in incongruent trials (*p* < 0.01), whereas there was no prominent difference in non-athletes’ RT between congruent and incongruent trials (*p* = 0.96). Additionally, neither an main effect of group, *F*_(1,51)_ = 2.79, *p* = 0.10, η_p_^2^ = 0.05, nor an interaction between load and congruency, *F*_(1,51)_ = 3.01, *p* = 0.09, η_p_^2^ = 0.06, was found.

Most importantly, a significant three-way interaction among group, load and congruency was identified, *F*_(1,51)_ = 10.21, *p* < 0.01, η_p_^2^ = 0.17 (Figure 4B). Simple effects analysis yielded a pronounced interaction in athletes’ RT between load and congruency, which was not observed in non-athletes’ RT. Specifically, at low load, athletes showed significantly faster responses in congruent conditions compared to incongruent conditions, *p* < 0.001. However, at high load, there was no significant difference between congruent and incongruent responses for athletes, *p* = 0.97. In contrast, non-athletes showed no significant difference in RT between congruent and incongruent conditions, neither under low-load (*p* = 0.33) nor high-load conditions (*p* = 0.58).

Regarding RA, a significant main effect of group was found, *F*_(1,51)_ = 5.52, *p* = 0.02, η_p_^2^ = 0.10, suggesting that athletes reacted grater than non-athletes. Furthermore, a significant main effect of congruency was identified, *F*_(1,51)_ = 9.25, *p* < 0.01, η_p_^2^ = 0.15, indicating that RA in congruent trials was higher than that in incongruent trials. In addition, no other main/interaction effect was significant.

Moreover, a significant four-way interaction among experiment, group, load and congruency was found in RT and RA, *F*_(1,98)_ = 4.02, *p* < 0.05, η_p_^2^ = 0.04, *F*_(1,98)_ = 4.02, *p* < 0.05, η_p_^2^ = 0.04, indicating that there was a significant three way interaction among group, load, and consistency conditions in Experiment 6, while there was not in Experiment 5.

#### 2.2.5 Discussion

The results of Experiment 2 demonstrated that when unconscious stimuli were related to sport scenarios, athletes displayed significantly larger unconscious priming effects under working-memory load compared to non-athletes. These findings are reconciled with extant research, which utilized materials relevant to real sporting contexts as the stimuli in the suppressed priming task and claimed superior unconscious processing in athletes. Kiesel et al. (2009), adopting chess configurations as masked stimuli, reported stronger unconscious priming effects in chess experts than in non-athletes. Similarly, Güldenpenning et al. (2015) introduced body postures from different moving stages as suppressed stimuli and showed that martial arts athletes showed larger subliminal priming effects than non-athletes. Moreover, table tennis athletes also displayed larger masked congruency effects than non-athletes when the subliminal stimuli were table tennis balls with hitting point (Meng et al., 2019; Shi et al., 2024). Although the aforementioned articles have emphasized a robust advantage in unconscious processing among athletes, to our knowledge, the current paper serves the first investigation into the advantage of unconscious priming in table tennis athletes under working-memory load.

Although athletes in Experiment 2 displayed stronger unconscious priming effects under working-memory load relative to non-athletes, it should be noted that their unconscious priming effects still decreased significantly with an increase in working-memory load. Considering that these athletes selected in our study have been extensively documented to demonstrate superior unconscious processing abilities than non-athletes (Geng et al., 2020; Meng et al., 2019, 2022; You et al., 2018), these findings suggest that even individuals with extensive subliminal experience have their unconscious processing impaired by working-memory load. This implies a universal dependence of unconscious processing on executive attention.

## 3 General discussion

The current research represents as a pioneering investigation into whether motor expertise modulates the effect of working-memory load on unconscious priming in athletes, and whether such modulation is influenced by the stimuli domain. To address these issues, experienced table tennis athletes completed a dual-task paradigm in which an N-back task was intermixed with a sandwich priming task. In Experiment 1, the stimuli in the priming task were domain-general, whereas in Experiment 2, domain-specific stimuli were employed. The findings confirmed our hypotheses that athletes displayed an advantage in unconscious processing under working-memory load when the unconscious stimuli were relevant to sport-specific realms rather than general realms. Moreover, increased working-memory load impaired unconscious priming effects in athletes and non-athletes regardless of whether the stimuli were sports-related. Therefore, this investigation strongly supports and extends the generalizability of the existing attention limitation hypothesis of unconscious processing (Ansorge et al., 2014; Martens et al., 2011; Hung et al., 2020) by demonstrating that unconscious priming in athletes still requires executive attention.

In Experiment 1, we found that when the masked stimuli were unrelated to sports scenarios, athletes did not perform better than non-athletes in the unconscious priming task under working-memory load. This result is consistent with earlier studies that employed arrows as suppressed stimuli and observed significant unconscious priming effects in both table tennis athletes and non-athletes (Meng et al., 2025; Jiang et al., 2024). However, one study applied general shapes as suppressed stimuli and showed that only table tennis athletes showed pronounced unconscious congruency effects (Geng et al., 2020). It is noteworthy that the latter study required participants to discriminate among four targets in the suppressed priming task, potentially enhancing task difficulty. However, the literature that reported significant unconscious priming effects in both athletes and non-athletes, including the present research and other studies (Meng et al., 2025; Jiang et al., 2024), all introduced two targets in the suppressed priming task, which might reduce the task difficulty. Prior research has indicated that athletes’ cognitive processing advantages are more significant under high cognitive demands (Meng et al., 2019; You et al., 2018). Thus, we suppose that the differences in task difficulty might account for the discrepancies between our observations in Experiment 1 and those of Geng et al. (2020).

The observations from Experiment 2 showed that, under working-memory load, athletes exhibited an advantage in sport-specific unconscious priming compared to non-athletes, supporting the view that specialized training can enhance unconscious processing performance (Güldenpenning et al., 2011, 2015; Kiesel et al., 2009). Based on extant research, athletes, particularly those from open-skill sports, are trained to process information in rapidly changing and unpredictable environments (Nakamoto and Mori, 2012; Nakamoto et al., 2013; Tamaki et al., 2017), which may contribute to their superior unconscious processing (Meng et al., 2019, Shi et al., 2024; You et al., 2018). This perspective is further supported by numerous studies providing functional magnetic resonance imaging (fMRI) and electroencephalographic (EEG)/ERP evidence. Chavan et al. (2015) have observed that short- or medium-term training can induce structural changes in the gray and white matter in the inferior frontal gyrus, which critically involved in unconscious priming processing (Shi et al., 2022). Furthermore, long-tern sports training alters the white matter microstructure of the fronto-basal response control network (Chavan et al., 2017), which is also implicated in unconscious processing (D’Ostilio and Garraux, 2012; Ulrich and Kiefer, 2016). Moreover, You et al. (2018) asked table tennis athletes to perform a masked go/no-go task and observed that shorter N2 latencies and larger no-go P3 effects in the fronto-central areas among athletes. These data indicate that long-tern training for fast motor reactions leads to changes in brain structure and neural networks, which may enhance their unconscious processing capabilities.

Remarkably, the two experiments showed distinct patterns of unconscious priming under working-memory load, implying that motor expertise and the stimuli domain play vital roles in unconscious processing under working-memory load. When the stimuli were domain-general and unrelated to sports experience, both athletes and non-athletes were sensible to these stimuli; therefore, fewer executive attention resources were required for their effective processing. Accordingly, both athletes and non-athletes displayed significant priming effects under low load. However, under high load, executive attention was completely depleted, which was insufficient for either group to process the stimuli effectively; thus, their priming effects were significantly reduced or even disappeared in Experiment 1. The observations of Experiment 1 were in line with the two-pool model of attention resources on the relationship between working memory and unconscious priming proposed by our prior work (Mao et al., 2022; Mao and Li, 2022). We have found in the prior work that an increase in working-memory load decreased the magnitude of unconscious priming in the manipulation dual task, whereas an increase in working-memory load did not decrease unconscious priming in the maintenance dual task. These observations demonstrate that the manipulation subsystem, rather than the maintenance subsystem, interferes with unconscious priming. When the stimuli were domain-specific and relevant to sports experience, athletes, rather than non-athletes, were sensible to these stimuli; therefore, athletes were able to effectively process such stimuli even with limited executive attention resources, whereas non-athletes were not. Consequently, under low load, although part of executive attention was consumed, the remaining resources were sufficient for athletes, but not for non-athletes, to process these stimuli effectively. Thus, in Experiment 2, athletes exhibited significantly larger priming effects than non-athletes across all load conditions. In summary, these findings not only highlight the effect of specialized training experience on unconscious priming under working-memory load but also emphasize the moderating role of stimuli domain in this relationship. Thereby, our study underscores the facilitative effect of long-term experience on unconscious processing under working-memory load, illustrates the boundaries of athletes’ advantages in unconscious processing under working-memory load, and provides deeper insights into the interactions between motor experience, unconscious processing, and working-memory load.

Our findings indicated that unconscious priming weakened with an increase in manipulation load in Experiments 1 and 2. These results are supported by numerous neurotechnological studies, claiming that unconscious processing requires high-level cognitive attention from the anterior brain cortex. By combining a masked oddball paradigm with ERP measurements, Silverstein et al. (2015) found that unconscious stimuli elicited significant oddball P3b effects and positive late slow wave effects. Moreover, a study utilizing a masked affective priming task with EEG methods observed that subliminal stimuli could lead to enhanced midfrontal theta activity and suppressed parieto-occipital alpha activity (Jiang et al., 2018). Furthermore, employing a masked go/no-go paradigm, previous studies reporteded enhanced oscillatory synchrony in the prefrontal-occipital cortex (Cohen et al., 2009) and robust theta priming effects in the fronto-central areas (Diao et al., 2021). In addition, studies integrating the subliminal visuomotor priming paradigm with event-related fMRI assessments have uncovered remarkable priming effects in both neural activity (D’Ostilio and Garraux, 2012) and functional connectivity within the fronto-parietal cortex (Ulrich and Kiefer, 2016). Shi et al. (2022) adopted activation likelihood estimation to exam fMRI studies and identified the essential involvement of the right inferior frontal gyrus in unconscious processing. Although these studies support that unconscious processing demands higher-level cognitive resources, our research directly demonstrates the dependence of unconscious processing on executive attention by manipulating the working-memory load. Therefore, this study, together with our previous work (Mao et al., 2022; Mao and Li, 2022), collectively emphasizes the demand for executive attention in unconscious processing, thereby deepening our understanding of the relationship between unconscious processing and resource requirements, and extending the existing frameworks of attention limitation hypothesis of unconscious processing (Ansorge et al., 2014; Hung et al., 2020; Kiefer et al., 2012).

Although our work addresses a gap in the extant literature and provides valuable insights into unconscious processing in athletes under working-memory load, some limitations warrant mention. First, the cross-sectional design adopted here may limit the establishment of a causality relationship between training experience and athletes’ unconscious processing advantages. Therefore, longitudinal intervention investigation should be conducted to more definitively link training duration and intensity with enhancements in unconscious processing capabilities under working-memory load. Second, although this research provides strong behavioral evidence supporting that motor expertise facilitates athletes’ abilities in unconscious processing under working-memory load, further investigation should explore the underlying neural mechanisms through multi assessments (e.g., EEG/ERP, fMRI, MEG). Third, the inclusion of only skilled table tennis athletes may limit the generalizability of the current findings to a broader population. Consequently, future research could integrate athletes from various sports disciplines to compare and validate the present results. Moreover, assigning fixed response hands (right for priming, left for memory) can induce motor interference or muscular imbalance over prolonged trials; therefore, we will implement hand counterbalancing across participants to eliminate potential motor bias and ensure response neutrality in the future studies. In addition, we need to conduct further experiments to examine whether the result discrepancies between the present study’s and Geng et al. (2020) are attributed to task difficulty. Finally, considering our relatively small sample size, subsequent research should enroll larger cohorts and consider individual differences to strengthen the robustness of the conclusions.

## 4 Conclusion

Taken together, this research represents the first investigation into the characteristics of unconscious processing in athletes under working-memory load. Utilizing a dual-task paradigm established in our prior work, in combination with subliminal stimuli from distinct domains and an expert-novice comparison, we observed that athletes exhibited larger unconscious priming effects than non-athletes under working-memory load when the subliminal stimuli were relevant to sport-specific domains rather than general ones. This observation suggests that athletes’ unconscious processing advantages under working-memory load may be limited to sports-specific contexts. Thus, our work deepens the understanding of the boundary conditions of athletes’ superiority in unconscious processing and contributes to enriching the literature on athletes’ advantages in information processing from the conscious realm to the unconscious realm. Furthermore, we found that even when subliminal stimuli were linked to sporting scenarios, athletes’ unconscious priming effects were still impaired with growing working-memory load. This result demonstrates that even athletes, who are identified to show advantage in unconscious processing, also require working-memory resources for unconscious processing. Therefore, our observations strongly support the attention gating theory of unconscious processing and extend its extant frameworks from non-athletes populations to skilled athletes.

## Data Availability

The raw data supporting the conclusions of this article will be made available by the authors, without undue reservation.

## Footnotes

1 “Unconscious processing,” “unconscious priming,” and “subliminal priming” discussed in this study refers to the processing of subliminal information that cannot be consciously accessed (Mudrik and Deouell, 2022), also known as subliminal processing (Dehaene et al., 2006). This type of unconscious does not involve states such as sleep or coma.

## Supplementary material

Supplementary Figure 1 The distribution of subjective reports on masking effectiveness (Perceptual Awareness Scale, PAS scores) in Experiment 1.

Supplementary Figure 2 The distribution of subjective reports on masking effectiveness (Perceptual Awareness Scale, PAS scores) in Experiment 2.
