# Supplementary figures and images for: Athletes demonstrate an advantage in unconscious processing under working-memory load specifically within sport-related domains
Source: Front Psychol. 2025 Nov 12;16:1697078. doi: 10.3389/fpsyg.2025.1697078 (PMC12647113; doi:10.3389/fpsyg.2025.1697078)

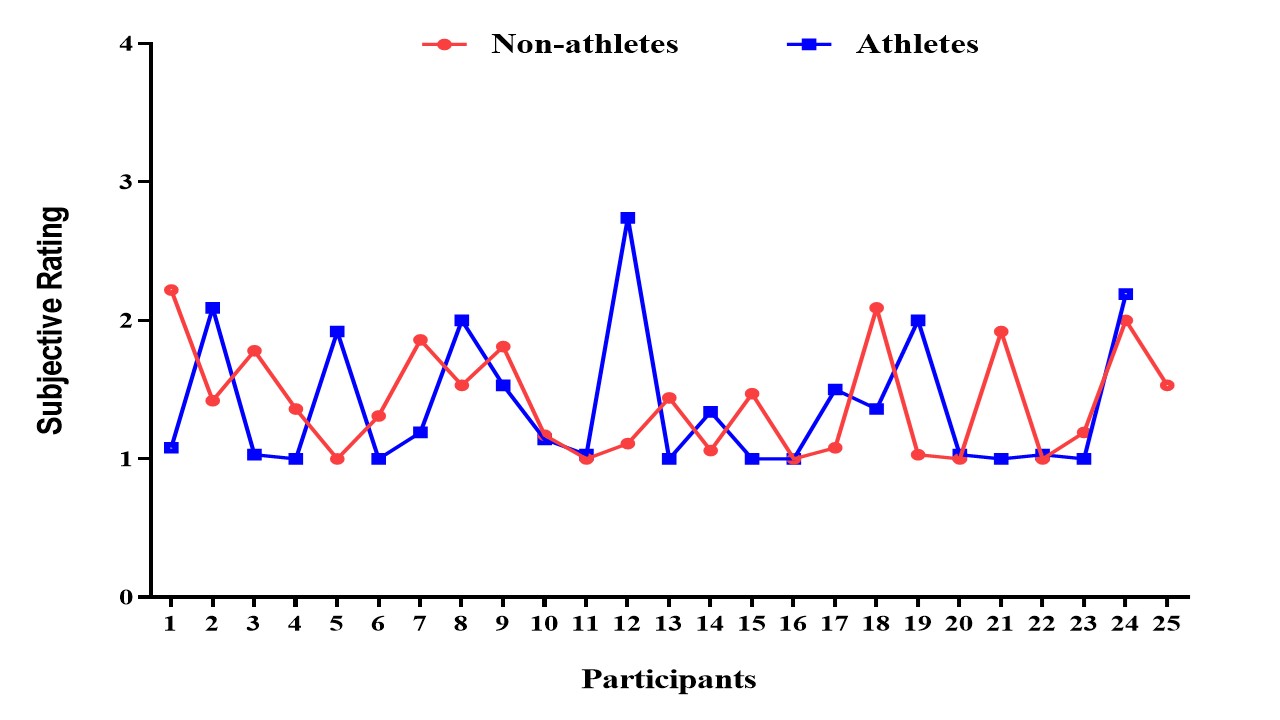

Supplement: Supplementary Figure 2 — The distribution of subjective reports on masking effectiveness (Perceptual Awareness Scale, PAS scores) in Experiment 2. [file Image_2.jpg]
